# Evolution of the SARS‐CoV‐2 omicron variants BA.1 to BA.5: Implications for immune escape and transmission

**DOI:** 10.1002/rmv.2381

**Published:** 2022-07-20

**Authors:** Lok Bahadur Shrestha, Charles Foster, William Rawlinson, Nicodemus Tedla, Rowena A. Bull

**Affiliations:** ^1^ School of Medical Sciences Faculty of Medicine UNSW Sydney New South Wales Australia; ^2^ The Kirby Institute UNSW Sydney New South Wales Australia; ^3^ Serology and Virology Division Department of Microbiology New South Wales Health Pathology Sydney New South Wales Australia

**Keywords:** immune evasion, monoclonal antibodies, omicron, SARS‐COV‐2

## Abstract

The first dominant SARS‐CoV‐2 Omicron variant BA.1 harbours 35 mutations in its Spike protein from the original SARS‐CoV‐2 variant that emerged late 2019. Soon after its discovery, BA.1 rapidly emerged to become the dominant variant worldwide and has since evolved into several variants. Omicron is of major public health concern owing to its high infectivity and antibody evasion. This review article examines the theories that have been proposed on the evolution of Omicron including zoonotic spillage, infection in immunocompromised individuals and cryptic spread in the community without being diagnosed. Added to the complexity of Omicron's evolution are the multiple reports of recombination events occurring between co‐circulating variants of Omicron with Delta and other variants such as XE. Current literature suggests that the combination of the novel mutations in Omicron has resulted in the variant having higher infectivity than the original Wuhan‐Hu‐1 and Delta variant. However, severity is believed to be less owing to the reduced syncytia formation and lower multiplication in the human lung tissue. Perhaps most challenging is that several studies indicate that the efficacy of the available vaccines have been reduced against Omicron variant (8–127 times reduction) as compared to the Wuhan‐Hu‐1 variant. The administration of booster vaccine, however, compensates with the reduction and improves the efficacy by 12–35 fold. Concerningly though, the broadly neutralising monoclonal antibodies, including those approved by FDA for therapeutic use against previous SARS‐CoV‐2 variants, are mostly ineffective against Omicron with the exception of Sotrovimab and recent reports suggest that the Omicron BA.2 is also resistant to Sotrovimab. Currently two new Omicron variants BA.4 and BA.5 are emerging and are reported to be more transmissible and resistant to immunity generated by previous variants including Omicron BA.1 and most monoclonal antibodies. As new variants of SARS‐CoV‐2 will likely continue to emerge it is important that the evolution, and biological consequences of new mutations, in existing variants be well understood.

**Abbreviations**
ACE2: Angiotensin converting enzyme
FCS: *Furin cleavage site*
mAb: Monoclonal antibody
NAb: Neutralising antibody
NTD: N‐terminal domain
ORF S: Open reading frame encoding the spike protein
RBD: Receptor binding domain
VOC: Variants of concern

## 1 INTRODUCTION

**FIGURE 1:**
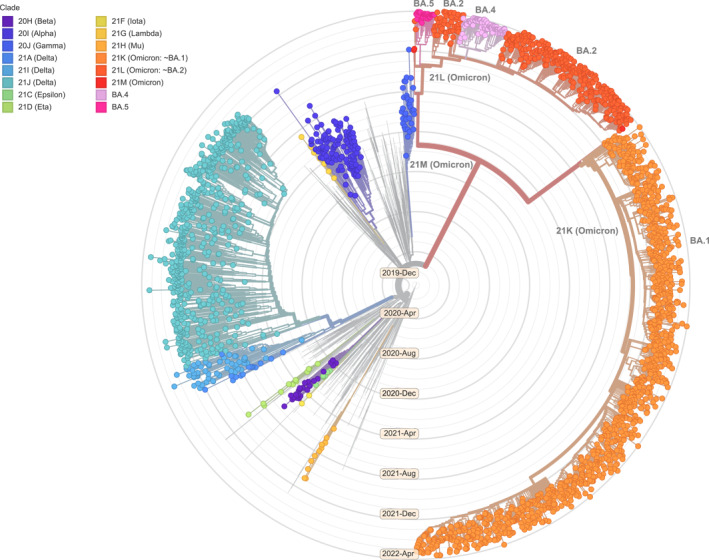
A time‐scaled phylogenetic tree of a representative global subsample of 3110 SARS‐CoV‐2 genomes, with tips coloured according to Nextstrain clades that predominantly correspond to variants of concern. Samples corresponding to Omicron sublineages BA.1, BA.2, BA.4, and BA.5 are labelled. BA.3 is not included since it does not yet satisfy the Nextstrain clade definition criteria; however, BA.3 falls under the overall Omicron clade 21M. Figure modified from the Nextstrain ‘omicron‐recombinant’ build (2022‐04‐08),^5^ using data available from the GISAID initiative (accession and author details available in Supplementary Table1).

Since the emergence of the SARS‐CoV‐2 virus in November 2019, several variants of concern (VOC) have emerged and rapidly spread with a global distribution.^1^ A variant is characterised as a VOC if it demonstrates increased transmissibility, virulence, change in disease presentation, or causes reduced effectiveness of vaccine induced protection, diagnostic tests and management measures.^2^ Chronic infection and co‐infection of an individual with different SARS‐CoV‐2 variants, and subsequent genome recombination play important role in the ongoing evolution of the SARS‐CoV‐2 variants.^3^, ^4^ Omicron is somewhat distantly related to previous VOCs (Figure 1),^5^ and is of significant public health concern since it carries several mutations that were also found in other VOCs and were associated with increased infectivity and enhanced capacity to evade the immune system.^6^

**FIGURE 2:**
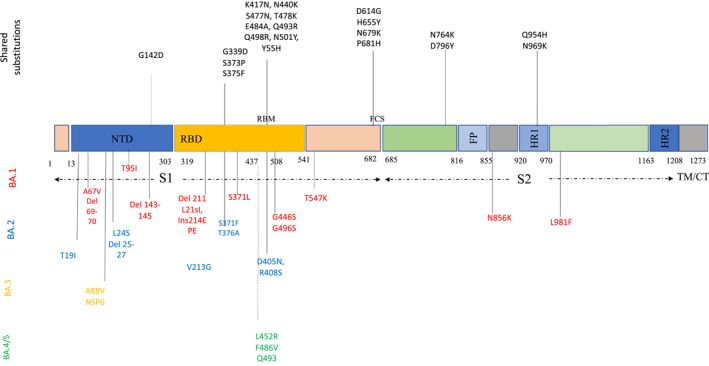
Amino acid substitutions within the Omicron variant lineage. Black colour represents shared mutations, red Omicron BA.1, blue BA.2, orange BA.3 and Green BA.4/BA.5. BA.4 and BA.5 share a similar spike profile as BA.2, except for additional mutations: 69‐70del, L452R, F486V (Green) and reversion to wild type: Q493 (Q493R in BA.1, BA.2 and BA.3). BA.4 and BA.5 differ from each other by 3 amino acid mutations outside Spike. BA.4 additional mutations: ORF7b:L11F, N:P151S. BA.5 additional mutations: M:D3N. Figure drawn using Microsoft Office PowerPoint.

**FIGURE 3:**
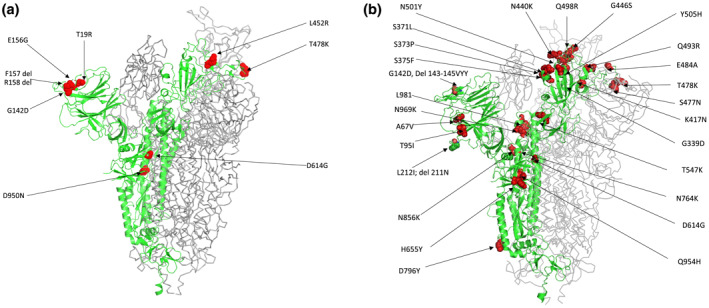
(a) Ribbon representation of Spike protein substitution in Delta variant (PDB ID 7W92), red spheres indicate amino acid substitution (b) Ribbon representation of Spike protein substitution in Omicron variant (PDB ID 7TGW), red spheres indicate amino acid substitution. Figure drawn by using Pymol; residues obtained from PDB (www.rcsb.com) using following PDB ID 7W92, 7TGW).

Omicron was first identified on mid‐November 2021, in South Africa and was designated as a VOC on 26^th^ November 2021.^7^, ^8^ Retrospective analysis revealed that Omicron was present in Europe 10 days before its discovery in South Africa with no obvious transmission link between the two locations.^9^ Compared to the Wuhan‐Hu‐1 reference genome, the Spike region of the originally described BA.1 Omicron genome had 35 mutations resulting in 30 amino acid substitutions, three in‐frame deletions, and an insertion of three amino acids (ins214EPE). 15 of these mutations fall in the receptor‐binding domain (RBD), a dominant binding site of the virus to the permissive host cells and a target of neutralising antibodies (NAbs)^6^, ^10^, ^11^ (Figure 2), hence they carry significant clinical relevance. The Omicron variant also harbours three and six mutations in regions coding for the membrane protein and the nucleocapsid protein, respectively.^12^ Many of the mutations within the Spike region of Omicron have been observed previously in other variants: del69‐70 was found in Alpha, T95I was present in Kappa and Iota, and G142D was present in Kappa and Delta^6^ (Figure 3).

Nine of the 15 RBD mutations in the Omicron Spike region fall in the binding footprint of the virus's main entry receptor, the human angiotensin‐converting enzyme (ACE2).^13^ Mutations within the RBD can potentially provide an evolutionary advantage by strengthening the viruses ACE2‐RBD binding, or by avoiding detection by NAbs.^14^, ^15^ Extrapolations based on observed mutations and preliminary data suggest Omicron will spread faster and evade antibodies more readily than earlier variants and thus increasing the chances of reinfection and breakthrough infections in the immunised population.^16^ In particular, Omicron carries some of the mutations responsible for the high infectivity of Delta, and it was believed that the reproductive number (*R*_0_) could increase to >30.^17^ Some estimates have indicated that Omicron BA.1 is three to six times more infectious than previous variants,^17^ with several countries reporting short doubling times^16^‐ 1.8 days (UK), 1.6 days (Denmark), and 2.0 days (United States).^18^ However, transmission and reproductive number of SARS‐CoV‐2 viruses depend upon several factors like social distancing, housing, ventilation, superspreading events and vaccination rates; therefore, it is difficult to directly correlate the observed transmission rate to variant phenotype.^19^, ^20^, ^21^

A striking feature of Omicron is that it comprises three distinct sub‐lineages (BA.1, BA.2, and BA.3) that were discovered near simultaneously, despite each sub‐lineage being as different from one another as Alpha, Beta, Gamma and Delta are from one another.^6^, ^22^ Subsequently, two other broad sublineages have been defined, BA.4, BA.5, as well as many sublineages within BA.1 and BA.2. Initially, BA.1 was the most prolific sub‐lineage detected worldwide; however, BA.2 (and its constituent sublineages) is overtaking BA.1 as the dominant variant globally.^23^, ^24^ BA.1 and BA.2 share many common mutations, but each also has unique mutations; BA.2 has additional 8 unique mutations not found in BA.1 and lacks 13 mutations that BA.1 does have^25^(Figure 2). More recently two new sub‐lineages, BA.4 and BA.5, were discovered in South Africa, and have since been detected in countries including Belgium, France, China, Botswana, Portugal, Germany and Australia.^26^ The most recent common ancestor of BA.4 and BA.5 is estimated to have originated in mid‐20 November21,^27^ coinciding with the emergence of the other lineages, for example, BA.2 in early November 2021. The BA.4 and BA.5 spike, is most closely related to BA.2. In addition to mutations in BA.2, BA.4 and BA.5 have the mutations 69‐70del, L452R, F486V and wild type amino acid at position Q493.^27^ BA.4 and BA.5 have similar mutational patterns in the 5′ genome region (from ORF1ab to Envelope) yet exhibit divergence in the 3′ region (from M to the 3′ genome end). It has been suggested that BA.4 and BA.5 may have diverged via a recombination event, with a breakpoint suggested between the E and M genes.^27^

The spread of Omicron will likely have important implications for current strategies to contain the SARS‐CoV‐2 pandemic and may require urgent public health interventions to limit transmission and reduce morbidity.^28^, ^29^ This review article is intended to analyse the evolution of variants, with a major focus on the Omicron variants, and summarise the neutralising capacity of sera from vaccinated or naturally infected individuals against these variants.

## 2 THEORIES ON THE EVOLUTION OF THE OMICRON VARIANT

The origin of Omicron remains unclear. Phylogenetic analysis of global SARS‐CoV‐2 sequences has not revealed any close intermediary sequences between Omicron and its closest relatives, therefore the pathway to the emergence of Omicron is unclear.^30^ The evolutionary analysis did not reveal any special mutational profile or frameshift event that could suggest that it descends from the Alpha, Beta, Delta or Gamma variants.^6^ The very long branch of the Omicron lineage in a time‐calibrated tree might reflect a cryptic and potentially complex evolutionary history.^31^ The enormously high number of mutations observed in Omicron relative to the other SARS‐CoV‐2 variants has raised a theory that the environment in which Omicron evolved may differ from other known VOCs.^30^, ^32^ Many mutations in Omicron were rarely reported among previous variants, leading to three prevalent hypotheses regarding its evolutionary history.^5^, ^33^

The first hypothesis is that Omicron could have ‘cryptically spread’ and circulated in a population with insufficient viral surveillance and sequencing.^32^, ^33^ Second, Omicron could have evolved in a chronically infected COVID‐19 patient, such as an immunocompromised individual who provided a suitable host environment conducive to long‐term intra‐host virus adaptation.^32^, ^33^ The third possibility is that Omicron could have accumulated mutations in a nonhuman host and then jumped into humans.^32^, ^33^, ^34^ Currently, the second scenario represents the most popular hypothesis regarding the proximal origins of Omicron.^32^, ^33^, ^35^ Although there is no definite evidence supporting this theory, several studies have reported that extensive viral mutations do occur in severely immunocompromised patients, including those with AIDS and cancer.^36^, ^37^, ^38^ Because Omicron was first assembled and reported in South Africa, it has been speculated that SARS‐CoV‐2 evolved rapidly in this setting because of the weakened immune system of more than 20% of the local population that is HIV infected.^39^ Some studies strongly advocate that the mutations in Omicron are acquired from a non‐human host.^30^, ^33^, ^34^ A recent study compared the molecular spectrum of the 45 pre‐outbreak Omicron mutations with the molecular spectrum for SARS‐CoV‐2 variants known to have evolved in humans (hSCV2). They found that the molecular spectrum of Omicron was completely different from hSCV2 spectrum which would point towards a non‐human origin. After comparing the molecular spectrum with coronaviruses that evolved in different hosts using a principal component analysis, they found that the molecular spectrum of pre‐outbreak Omicron mutations was within the mouse ellipse, suggesting that the pre‐outbreak mutations accumulated in a rodent (in particular a mouse) host. They also showed that mutations in the open reading frame encoding the spike protein (ORF S) of pre‐outbreak Omicron share the same positions as the ORF S mutations identified in mice, not in the variants identified in human.^33^ These latter observations point to towards potential evolution within rodents.^30^ It is possible that an earlier variant of SARS‐CoV‐2 could have acquired mutations that increased its potential to infect rodents from an ill person likely through contaminated sewage leading to its evolution into Omicron in the rodent population.^32^

Improved understanding of the origin of Omicron, and any future VOCs, may thus require genomic surveillance of non‐human animals, particularly rodents, because of their potential role as intermediate hosts of SARS‐CoV‐2.^30^ Further evolutionary analysis of the ancestral SARS‐COV‐2 variant to Omicron may give us more clues about the exact origin of the Omicron variant.

## 3 IS OMICRON MORE INFECTIOUS BUT WITH LESS SEVERE DISEASE OUTCOMES?

Upon encountering a host cell, the surface bound Spike protein subunit S1 binds to the ACE‐2 receptor on the cell surface, and then S2 mediates membrane fusion for viral entry into the cell. Substitutions in the receptor‐binding domain of Omicron, such as Q493R, N501Y, S371L, S373P, S375F, Q498R, and T478K have conferred higher binding affinity to ACE2.^40^, ^41^ The furin cleavage site (FCS), located at the junction of S1 and S2, plays a key role in the fusion of the virus with the host cell.^42^ P681H has already been shown to enhance Spike cleavage in the Alpha and Delta (P681R), and Omicron contains 3 substitutions (N679K, H655Y, and P681H) close to the furin cleavage site. The 15 RBD and 3 furin cleavage site substitutions in Omicron suggest a major change in the infectivity is likely.^14^, ^43^

A recent study, using an artificial intelligence‐based model, predicted that Omicron BA.1 would be 10 times and 2.8 times more infectious than Wuhan‐Hu‐1 and Delta variant, respectively, mainly due to its RBD mutations N440K, T478K, and N501Y.^14^ An in vitro study reported that Omicron BA.1 pseudovirus infects 293T‐ACE2 cells 4‐fold more efficiently than Wuhan‐Hu‐1 pseudovirus and 2‐fold more efficiently than Delta.^11^ In another study, scientists studied the bronchus and lungs of SARS‐CoV‐2 patients infected with different variants and reported that Omicron BA.1 replicated approximately 70 times higher than the Delta and Wuhan‐Hu‐1 variant in the bronchus. In contrast, the replication was less efficient (more than 10 times lower) in the human lung tissue compared to Wuhan‐Hu‐1 SARS‐CoV‐2 virus, which they hypothesised would indicate that Omicron should cause less severe disease associated with the lower respiratory tract.^44^

Several epidemiological studies have verified the higher infectivity of Omicron BA.1 as compared to wild type. A study from Denmark compared household infection of Omicron BA.1 to Delta and found 1.17 times higher secondary attack rate in unvaccinated, 2.61 times in fully vaccinated and 3.66 times higher in booster‐vaccinated individuals, concluding strong evidence of immune evasiveness of Omicron.^18^

Despite Omicrons higher transmissibility, it has been suggested that it causes less severe disease. Two studies have modelled the effects of undocumented previous infections to estimate Omicron's intrinsic severity relative to Delta. Each study estimated that Omicron BA.1 was about 75% as likely as Delta to cause hospitalisation in an unvaccinated person with no history of SARS‐CoV‐2 infection.^45^, ^46^ A large scale study from South Africa among 131,628 people also concluded that people infected with Omicron BA.1 variant have lower odds of severe infection and hospitalisation as compared to Delta and other variants.^47^ In contrast, however, a USA based study that analysed state‐level vaccination data and hospitalisation data across different SARS‐CoV‐2 waves in over 130,000 patients. They concluded that hospitalisation and mortality risks were identical between the waves and inferred that Omicron might be as severe as previous variants.^48^ But the majority of studies do still suggest that infection with Omicron is associated with substantially reduced risk of progression to severe clinical outcomes, hospitalisation and death relative to Delta (B.1.617.2) variant.^49^, ^50^, ^51^, ^52^, ^53^, ^54^ However, the lower incidence of hospitalisation and deaths with Omicron compared to previous variants, is confounded by the high level of vaccination and previous infection by other variants that may confer some protection.^55^ A recent WHO report also showed, after adjusting for the confounding effects of age, sex, ethnicity, prior infection, vaccination status, comorbidities, effect of province and effect of public/private sector, evidence of reduced severity and lower mortality for the Omicron variant as compared with the Delta variant.^56^ Some studies also suggest that, among vaccinated individuals, in addition to milder infections in Omicron, the symptoms are shorter (6.87 vs. 8.89 days in 2‐dose vaccinated and 4.4 vs. 7.7 days among boosted) compared to the Delta variant.^50^, ^57^ It is worth noting that the clinical severity and mortality of SARS‐COV‐2 infections do not solely depend upon the infecting variant. Population level immunity and vaccination rate, population density, socio‐political factors and seasonality play a significant role.^20^, ^58^

Animal studies do support that Omicron is less likely to cause severe symptoms. Bentley et al. investigated the infectivity and severity of Omicron, in mouse models containing human ACE‐2 (k18‐hACE2 mice), with the wild‐type and Delta variants and found that mice infected with the Omicron variant had less severe clinical signs, showed faster recovery and had a reduced viral load in both the upper and lower respiratory tract.^59^ Moreover, studies investigating the cause behind the reduced severity of Omicron variant have concluded that Omicron has a reduced ability to induce syncytia in tissue culture. This is clinically significant because syncytia formation has been linked with heightened disease severity.^60^, ^61^, ^62^

Interestingly, an in vitro study using human nasal epithelial cells has suggested that BA.2 Omicron sub‐variant is 1.5 times more contagious than BA.1. BA.2 also showed significantly more cell fusion and 1.52‐fold larger syncytia than BA.1.^63^ The recent reports of increasing frequency of BA.2 in the context of the BA.1 surge are probably related to increased transmissibility rather than to enhanced immunologic escape.^25^ There has been no report to date of BA.2 being more clinically severe than the BA.1 lineage.^23^ Early data suggests that the recent sublineages of Omicron, BA.4 and BA.5 seem to have a growth advantage over the BA.1 and BA.2 variants, but why is not currently understood.^27^ This may be due to improvements in its intrinsic transmissibility or perhaps enhanced immune evasion with the F486V mutation.^27^

Overall, the infectivity of Omicron is much higher than the ancestral SARS‐CoV‐2 variant and other subsequent variants including Delta, mostly owing to its huge number of mutations in RBD and FCS.^14^, ^64^ However, fortunately real‐world data shows that the severity of illness hospitalisation and deaths in the Omicron wave is lower than preceding waves, whether this is due to lower pathogenesis of Omicron or protection from pre‐existing immunity is difficult to discern, but is a promising sign as we move towards living with COVID‐19.^14^, ^47^

## 4 ESCAPE OF OMICRON FROM IMMUNITY AGAINST PREVIOUS COMMUNITY INFECTION BY OTHER VARIANTS

With the emergence of new variants, a key question always remains ‐ will this variant escape pre‐existing immunity generated to a previous variant? A large‐scale study in South Africa conducted during initial period of Omicron BA.1 rise showed clear evidence of population‐level immune escape. The number of daily new reinfections, which was not evident during the circulation of the Alpha and Delta variant, has spiked with Omicron and exceeded the 95% projection accompanied by a dramatic increase in the hazard ratio for reinfection versus primary infection.^65^ The authors, however, cautioned this was an epidemiological‐based study and laboratory neutralisation tests are ongoing to confirm this.

A study from Qatar revealed that effectiveness of previous infection in preventing reinfection was 90.2% against the Alpha variant, 85.7% against the Beta variant, 92.0% against the Delta variant, and 56.0% against Omicron BA.1.^66^ This suggests that the previous infection with other variants has considerably less protection against Omicron. However, protection was preserved against severe infection resulting in hospitalisation and death, regardless of the variants.^66^ One report estimates that the risk of reinfection with Omicron BA.1 is 5.4 times greater compared to the Delta variant. Pre‐Omicron, prior infection afforded 85% protection against a second COVID‐19 infection over 6 months; however, the protection against reinfection risk has fallen to 19% against Omicron infection.^46^

Researchers in one study attempted to study the neutralising titre of sera collected early in the pandemic against Omicron BA.1. Compared with wild‐type, the neutralisation titres of sera for Omicron BA.1 were reduced for early pandemic (16.9‐fold), Alpha (33.8‐fold), Beta (11.8‐fold), Gamma (3.1‐fold), and Delta (1.7‐fold). Omicron causes widespread escape from neutralisation by serum obtained following infection by a range of SARS‐CoV‐2 variants, meaning that previously infected individuals will have little protection from infection with Omicron.^13^ In recent research, investigators studied the neutralisation capacity of the Omicron BA.4 and BA.5 sub‐lineage from individuals infected by Omicron BA.1. The neutralisation of BA.4 and BA.5 was reduced by 7 fold among unvaccinated individuals and 3 fold in vaccinated individuals. This suggest that even the infection with previous Omicron sub‐lineage is unlikely to protect from upcoming Omicron sub‐lineages and these variants BA.4 and BA.5 are likely to cause new wave of infections.^67^ As BA.2 has recently caused a number of infections across Europe, it is hoped that this may confer better protection than BA.1 and studies are underway.

These data suggest that protection from previous natural infection has fallen greatly against Omicron variant and susceptibility to infection with Omicron and its sublineages is likely even in previously infected people.

## 5 IMMUNE ESCAPE BY OMICRON FROM NEUTRALISATION ANTIBODIES PRODUCED IN RESPONSE TO VACCINATION

The current COVID‐19 vaccines in use primarily target the S protein.^68^ The multiple mutations and deletions in the Spike protein of the Omicron variant render a part of the Spike protein unrecognisable to the antibodies raised by natural infection or vaccination indicating a strong capability of Omicron to evade humoral immune responses.^14^ Several studies have been conducted to measure the neutralising capacity of vaccine induced immunity against the Omicron variant, with all studies showing a marked reduction in neutralising capacity to the Omicron variant (8–127 times reduction in vaccine efficacy) (Table 1). Several studies have shown that with a booster shot vaccine effectiveness can be improved by 10–127 times (Table 1). A study, however, has shown that even with 3 doses of a mRNA vaccine, the vaccine induced immunity was only 66.3% effective against Omicron as compared to 88.5% against the Delta variant.^69^ Two mutations within the RBD, K417N (also seen in the Beta variant) and E484A are believed to be driving Omicron to cause a greater number of vaccine breakthroughs. Although reports to‐date suggest breakthrough infections have been mild, with severe infections rare in fully vaccinated individuals.^14^, ^29^ Moreover, the newer subvariants, BA.4, and BA.5 substantially escape neutralising antibodies induced by both vaccination and infection. NAb titres against the BA.4 or BA.5 subvariant are lower than titres against the BA.1 and BA.2 subvariants, which suggests that the SARS‐CoV‐2 omicron variant has continued to evolve with increasing neutralisation escape.^70^, ^71^

**TABLE 1:** Neutralisation potency of COVID‐19 vaccines against Omicron variant

Type of vaccine	Neutralisation assay	Efficacy against omicron after 2^nd^ dose	Days after booster	Increased omicron BA.1 neutralisation after booster (fold increase)
Zhang et a.^124^	Pseudovirus neutralisation test	Vs. ancestral strain: BNT162b2: ↓ × 8	6–69	BNT162b2: ↑ × 10
Garcia‐beltran^11^	Pseudovirus neutralisation assay	Vs. ancestral strain: BNT162b2: ↓ × 43; mRNA‐1273: ↓ × 122	<90	BNT162b2 ↑ × 27 mRNA‐1273: ↑ × 19
Haveri et al.^125^	Pseudovirus neutralisation assay	Vs. ancestral strain: BNT162b2: ↓ × 19.7	28	BNT162b2: ↑ × 38.4
Nemet et al.^126^	Live virus neutralisation assay	Vs. ancestral strain: BNT162b2: ↓ × 14.9	25	BNT162b2: ↑ × 96.9
Gruell H et al.^99^	Pseudovirus neutralisation assay	Vs. ancestral strain: BNT162b2: ↓ × 68.2	21	BNT162b2: ↑ × 132.8
Yu et al.^127^	Pseudovirus neutralisation assay	Vs. ancestral strain: BBIBP‐CorV: ↓ × 20.1	28	BBIBP‐CorV: ↑ × 3.3
Muik et al.^100^	Pseudovirus neutralisation assay	Vs. ancestral strain: BNT162b2: ↓ × 22.8	28	BNT162b2: ↑ × 23.4
EDara et al.^107^	Live‐virus focus reduction neutralisation test (FRNT)	None of the vaccinated had neutralising antibody titre after 6 months	7–28	90% of the subjects retained nAb titre
Schmidt et al.^128^	Pseudovirus neutralisation assay	Vs. ancestral strain: BNT162b2: ↓ × 127	30	BNT162b2: ↑ × 42.1
Mallory et al.	hACE2 receptor‐binding test	Vs. ancestral strain: NVX‐CoV2373: ↓ × 8.2	28	NVX‐CoV2373: ↑ × 14.8
Doria‐Rose et al.^98^	Pseudovirus neutralisation assay	Vs. ancestral strain: mRNA‐1273: ↓ × 8.9	14	mRNA‐1273: ↑ × 12.6

Research from the Oxford Vaccine group has suggested that Omicron variant BA.1 is more antigenically distant from the original SARS‐CoV‐2 vaccine strain than the previously most distant strains, Beta and Delta.^72^ This raises the question of what the best strategy is to combat new variants and whether it will be necessary to produce vaccines tailored to Omicron; however, these variant specific vaccines may not give protection against other variants. Researchers from Taiwan developed a panel of mRNA‐LNP based vaccines using RBD of Omicron, Delta and a hybrid. Omicron‐specific and hybrid vaccines produced high titre of NAbs against Omicron itself, but few to none against other variants.^73^ This therefore raises concerns about moving towards a variant specific vaccine as it is unknown if future variants will emerge from the Omicron lineage or from the Delta lineage, which still continues to circulate at lower frequency.^72^, ^73^ Also new variants have been emerging every 6 months so it is unlikely that new variant vaccines could be developed and distributed in a timely manner. Although preliminary, not yet peer‐reviewed, data from a recent Moderna vaccine trial that mixed ancestral Wuhan‐like variant with an Omicron BA.1 variant, indicated that this vaccine strategy can effectively induce broad neutralising responses. Hence further research into vaccine approaches is needed.^74^

## 6 IMMUNE ESCAPE BY OMICRON FROM THERAPEUTIC MONOCLONAL ANTIBODIES

Monoclonal antibody therapy has been highly effective at preventing hospitalisation and death, but the emergence of Omicron variant poses a major threat to the efficacy of current treatments.^40^ The majority (>90%) of the potent neutralising monoclonal antibodies characterised to date bind the RBD of the viral spike protein while some of them bind to N‐terminal domain (NTD). As a result, any mutation on the RBD and or NTD may cause immediate concerns about the efficacy of the existing mAbs.^40^, ^75^, ^76^ The Omicron variant contains a handful of mutations within the RBD that were previously considered to be highly conserved and are the target of monoclonal antibodies (Table 2).

**TABLE 2:** Efficacy of monoclonal antibodies against Omicron variant

Monoclonal antibodies	Antibody class	Efficacy against ancestral wild‐type variant	Efficacy against Delta	Efficacy against omicron BA.1	Contributing substitutions	
REGN10933 (casirivimab)	1	++	++	Not able to neutralise	K417N, E484A, S477N, Q493R	^10^, ^13^, ^82^, ^129^, ^130^, ^131^
REGN10987 (imdevimab)	3	++	++	Not able to neutralise	G446S	^10^, ^82^, ^129^, ^130^, ^131^
					N440K	
Eli Lily Estevimab (LYCoV16)		++	+	Not able to neutralise	S477N,	^82^, ^129^, ^131^
					K417N,	
					Q493R	
LYCoV‐555 (bamlanivimab)	2	++	‐	Not able to neutralise		^31^, ^82^, ^130^
CT‐P59		++	+	Do not neutralise	K417N,	^129^
					E484A,	
					Q493R, G496S, Q498R	
GSK and virSotrovimab/S309	3	++	+++	2–3 fold reduction compared to Wuhan‐Hu‐1	G339DN44oK	^13^, ^82^, ^129^, ^131^
AstraZeneca Evusheld Cilgavimab/tixagevimab	2 and 1	++	++	Retain neutralising titre	T478K, Q493R, S477N, G446S	^13^, ^82^, ^129^, ^131^
					E484A	

Whereas the previous VOCs displayed substitutions only in the epitope targeted by class 1 and 2 mAbs, the Omicron mutations are situated within the binding site of all four epitopes targeted by mAbs.^31^ Among the 15 RBD substitutions in the Omicron variant, the K417N substitution, which is also carried by the Beta variant, is responsible for the most significant disruption to the known mAbs.^14^ Several mAbs interact with E484 on the SARS‐CoV‐2 RBD, but the E484A substitution is unfavourable leading to antibody escape.^13^ The substitutions S371L, S373P and S375F form part of the class 4 epitope, affecting previously described class 4 antibodies such as Ab‐3467 that broadly neutralise sarbecoviruses.^77^, ^78^ The class 3 antibody Sotrovimab (mAb S309) targets a highly conserved region of the sarbecovirus RBD^40^, ^79^ and retains neutralising potency against Omicron BA.1, despite two mutations within its epitope (G339D & N440K).^80^

Recent reports suggest that Omicron BA.2 causes more antibody evasion than BA.1 owing to the additional S371F, T376A, D405N and R408S substitution.^81^, ^82^ It has been reported that BA.2 exhibited marked resistance to 17 of 19 neutralising mAbs tested.^81^ Interestingly, even Sotrovimab that had appreciable effects against BA.1, lost 27‐fold neutralising activity against BA.2.^81^ A study, however, reported that the mAb cocktail Evusheld (Cilagavimab and Tixagevimab) can neutralise BA.2 better than BA.1.^83^ Another research study reported that reduction in neutralising capacity of mAbs against Omicron BA.2 was less compared to BA.1 and BA.3,^84^ suggesting that the neutralisation could also depend upon individual mAbs, and might be too soon to predict if BA.2 is more resistant to antibodies than BA.1. The new Omicron sublineages‐ BA.4 and BA.5‐ have been reported to impart even higher resistance against the broad mAbs than BA.1 and BA.2.^85^, ^86^ Pseudoviruses harbouring the spike of these newer Omicron variants (BA.4 and BA.5) were tested for their neutralisation sensitivity against a range of therapeutic mAbs. Most of the mAbs tested failed to neutralise BA.4 and BA.5; however, interestingly these variants were more sensitive to sotrovimab than BA.2.^85^ Cao et. al. suggested that, with the exception of Babtelovimab and Cilgavimab, the subvariants BA.4 and BA.5 are resistant to most broad nAbs.^86^ These findings have been similarly reported in other studies.^70^ The evasion is attributed to several substitions, in particular, S371F, D405N, R408S, F486 and L452R.^85^, ^86^

Taken together, the Omicron variant and its sublineages completely or partially escape neutralisation by the tested antibodies. There are several mutations in the RDB and NTD of Omicron variant making them unrecognisable to several mAbs. Most of the therapeutic antibodies are unable to treat Omicron infected patients; however, mAbs like Sotrovimab and AstraZeneca cocktail still retain some neutralising activity against the BA.1. With the reports of BA.2 becoming resistant to the highly effective Sotrovimab, it might be an uphill task in treating severe infections since there are no therapeutic antibodies effective against all SARS‐CoV‐2 variants. It is high time to focus research and development of newer mAbs that can neutralise the newer variants including Omicron.

## 7 OMICRON DOES NOT APPEAR TO ESCAPE T CELL RESPONSES TO OTHER SARS‐COV‐2 VARIANTS

The role of T cells in protection against SARS‐CoV‐2 has been well established.^87^, ^88^ SARS‐CoV‐2 T cell responses induced by either natural infection or vaccines have been linked to rapid viral clearance and reduced disease severity,^87^, ^89^ even when the NAb response is reduced^90^ or absent.^91^ A study conducted in the USA reported that almost all individuals with existing anti‐SARS‐CoV‐2 CD8+ T‐cell responses were able to recognise the Omicron variant suggesting this variant has not evolved extensive T‐cell escape mutations at this time.^92^

Data from La Jolla Institute has revealed that, despite several mutations in S protein, on average 94% of CD8 and 91% of CD4 epitopes are still completely conserved and this was supported by another study that indicated that only 14% of CD8^+^ and 28% of CD4^+^ T cell epitopes contain at least one position harbouring an Omicron mutation, suggesting that the majority of CD8^+^ and CD4^+^ T cell epitopes still remain unaffected by Omicron.^93^ The frequencies of SARS‐CoV‐2 spike‐specific CD4+ T cells that cross‐recognized Omicron in natural infected or BNT162b2‐vaccinated individuals were 84% and 91%, respectively, and for CD8+ T cells were 70% and 92%, respectively. The data suggest that established SARS‐CoV‐2 Spike‐specific CD4+ and CD8+ T cell responses remain largely intact against Omicron.^94^ These findings and clinical data suggest that T cells are largely unaffected against this Omicron variant and may be a key component in helping keep severe disease at bay.

## 8 EVIDENCE OF IMPROVED PROTECTION AGAINST OMICRON BY BOOSTER VACCINATION

With the reports of waning NAb response months after the second dose of COVID‐19 vaccine,^95^, ^96^, ^97^ coinciding with the emergence of Omicron variant, the effectiveness of the booster shot against the Omicron variant has been closely examined. Several studies have shown that despite the nAb response against Omicron BA.1 being minimal after the complete two dose vaccination schedule, after the booster shot, the NAb titre against Omicron BA.1 was significantly improved by 12–35 fold^13^, ^98^, ^99^, ^100^, ^101^ (Table 2).

An established statistical model,^102^ utilising the previously published clinical data,^10^, ^103^, ^104^ predicted that the efficacy of prior mRNA vaccination against Omicron variant will wane to 40% against infection and 80% against severe disease. However, a booster dose with an existing mRNA vaccine (even though it targets the ancestral Spike) has the potential to raise efficacy against Omicron to 86.2% against symptomatic infection and 98.2% against severe infection.^105^

The Omicron subvariants BA.4/BA.5 have shown accentuated resistance against Nabs elicited by natural infection or vaccination.^70^, ^71^, ^86^ But booster vaccination has been reported to provide sufficient neutralising‐antibody titres against the BA.four‐fifths, albeit to a lower extent than against BA.1 and BA.2.^101^, ^106^

The administration of a booster dose has been particularly good at reducing severe illness, and hospitalisation with Omicron variant infections. The neutralising antibody response which usually wanes within months after the second dose,^95^, ^107^ is usually restored after the introduction of a booster. With the booster dose providing some additional protection against Omicron variant, the scientific world is curious about the possibility of a fourth dose of a vaccine. However, studies from Israel suggested that a fourth dose of a COVID‐19 vaccine restores antibodies to levels observed after the third dose but provides only a modest short‐term boost in protection against infection.^108^, ^109^ The fourth dose might be beneficial for immunocompromised individuals but may not be practical and sustainable for everyone.

Heterologous combinations of spike encountered during infection and vaccination shape subsequent cross‐protection against VOCs. Because heterologous combinations can confer a diminished response against other variants due to immune imprinting, there may be a case for sticking with the WuhanHu‐1 sequence in booster vaccinations.^110^ Previously infection‐naïve HCW who became infected during the B.1.1.529 wave showed enhanced immunity against earlier variants, but reduced nAb potency and T cell responses against B.1.1.529 itself.^111^ Recently, both Pfizer and Moderna has introduced booster including the S protein of Omicron variant with claims of better protection against Omicron variant.^112^, ^113^ The efficacy in the real world population is yet to be seen.

## 9 THE POTENTIAL EPIDEMIOLOGICAL AND CLINICAL SIGNIFICANCE OF OMICRON RECOMBINANTS

Recombination is an important source of variation for most viruses.^114^ The process of viral recombination is important for public health, since it can lead to factors such as increased virulence and pathogenicity, evasion of host immunity, and reduced effectiveness of vaccines and antivirals.^114^ Therefore, it is important to regularly screen for recombinant SARS‐CoV‐2 viruses.

For a significant portion of the COVID‐19 pandemic, there was no strong evidence for recombinant SARS‐CoV‐2 viruses despite widespread genomic surveillance efforts.^115^ Some early putative recombinants were the result of contamination and/or mixed infections within hosts.^116^, ^117^ Although it should be noted that the limited variation in the genomes in circulation early in the pandemic does make it difficult to confidently discern a true recombinant event versus convergent evolution. The first true recombinant pango lineage to be recognised, between the parental lineages of B.1.1.7 and B.1.177, was assigned the lineage ‘XA’.^116^ While XA was first designated in May 2021, the earliest date of collection of a sequence assigned to this lineage dates to 18 20 December20.^116^ Subsequently, a further 18 recombinant lineages have been recognised^116^ (Table 3). The majority of these recombinant SARS‐CoV‐2 lineages have arisen after the appearance of Omicron and have resulted from recombination between BA.1 and BA.2 and their associated sublineages (https://cov‐lineages.org/lineage_list.html).

**TABLE 3:** Recombinant lineages of SARS‐COV‐2

Lineage	Countries detected	First detected	Recombination between
XA	UK, US, Czech Republic, Sweden, Switzerland	2020‐12‐18	B.1.1.7 and B.1.177, UK lineage
XB	US, Mexico, Guatemala, Honduras,	2020‐07‐08	B.1.634 and B.1.631
XC	Japan	2021‐08‐12	AY.29 and B.1.1.7
XD	France, Denmark		Delta and BA., France and Denmark lineage
XE	Australia, UK		BA.1 and BA.2, UK lineage
XF	UK		Delta and BA.1
XG			BA.1 and BA.2, Denmark lineage
XH			BA.1 and BA.2, Denmark lineage
XJ			BA.1 and BA.2, Finland lineage
XK			BA.1 and BA.2, Belgium lineage
XL			BA.1 and BA.2, UK lineage
XM			BA.1.1 and BA.2, European lineage
XN			BA.1 and BA.2, UK lineage
XP			BA.1.1 and BA.2, UK lineage
XQ			BA.1.1 and BA.2, UK lineage
XR			BA.1.1 and BA.2, UK lineage
XS	USA		Delta and BA.1.1, USA lineage

Prior to the emergence of Omicron, the majority of worldwide COVID‐19 cases were caused by Delta, which was generally considered to cause more serious illness than Omicron.^14^, ^29^ Accordingly, potential recombinants between Omicron and Delta have been of concern to scientists and have received (arguably disproportionate) media coverage with the colloquial name ‘Deltacron’. The earliest putative cases of Deltacron were discredited as clear examples of laboratory contamination.^118^ However, there are now three lineages that are recognised as true recombinants between Omicron and Delta: XD (found in France and Denmark), XF (found in the UK), and XS (found in the USA).^119^, ^120^, ^121^, ^122^ WHO has recently added XD under variant under monitoring category.^2^ Despite early fears, there is no evidence yet that any of these ‘Deltacron’ variants have a greater infectivity than Omicron, reduced vaccine efficacy relative to Omicron, nor a greater clinical severity than Delta.^123^ Nevertheless, ongoing surveillance of recombination between lineages of Omicron, or between Omicron and other distantly related lineages, is warranted.

## 10 CONCLUSIONS

Omicron now has a foothold in many countries. It has an estimated doubling time of 2.5 days and 2 doses of vaccine appear to give low protection from infection, whereas 3 doses give better protection. Omicron variant is more infectious as compared to Wuhan‐Hu‐1 and Delta variant but severity appears to be less and may be associated to reduced syncytia formation. Antibody evasion is 40–80 fold higher in the Omicron variant as compared to the Wuhan‐Hu‐1 variant and Delta variant. However, T cell immunity is less affected by the mutations in the Omicron variant and likely remains key to protection against them. Although concerningly, Omicron spike is resistant to most therapeutic antibodies but it does remain susceptible to Sotrovimab, although Sotrovimab is less effective against the emerging BA.2 variant. The only viable option currently to control the spread of Omicron, barring social distancing and mask‐wearing, is to pursue vaccination with Wuhan‐Hu‐1 containing antigen including the booster dose. Widespread vaccine breakthroughs may mandate the production of a vaccine specific to Omicron. The increasing prevalence of the BA.2 sub‐lineage of Omicron in Europe and US and the increasing emergence of BA.4 in South Africa, along with the sporadic reports of the hybrid Deltacron show that the pandemic is not over and that we can expect to see the virus circulating at high levels. It is very hard to predict from here where the new antigenic variants will emerge ‐ Delta, Omicron, Deltacron, a new lineage, or whether multiple lineages may continue to circulate similar to influenza A and B.

## AUTHOR CONTRIBUTIONS

Lok Bahadur Shrestha and Rowena Bull conceived the work. Lok Bahadur Shrestha and Rowena Bull wrote the original draft. Lok Bahadur Shrestha and Charles Foster made the figures. Rowena Bull, Nicodemus Tedla, Charles Foster and William Rawlinson supervised and reviewed the manuscript. All authors read the final version of the submitted manuscript.

## CONFLICT OF INTEREST

No conflict of interest declared.

## Supporting information

Supporting Information S1

## Data Availability

The data used for generating Figure 1 is available as supplementary file.
